# Phylogenetic and Developmental Constraints Dictate the Number of Cusps on Molars in Rodents

**DOI:** 10.1038/s41598-019-47469-x

**Published:** 2019-07-29

**Authors:** Robert W. Burroughs

**Affiliations:** 1 Committee on Evolutionary Biology, The University of Chicago, Chicago, IL USA; 2 Integrative Research Center, Field Museum of Natural History, Chicago, IL USA

**Keywords:** Evolution, Evolutionary developmental biology, Evolutionary theory

## Abstract

Mammal tooth morphology and function correlate strongly with dietary ecology, and convergence is a major feature of mammalian tooth evolution. Yet, function and ecology are insufficient to explain morphological diversification and convergence within mammalian molar evolution; suggesting that development and phylogeny also limit possible structural solutions to selective pressures. Here, I use *in silico* models and empirical studies of extant and fossil rodent teeth to identify morphogenetic rules that influence molar morphology. Because rodents are the most diverse group of mammals with corresponding dental disparity they represent an excellent system for investigating how genetic interactions limit morphology. I find that lower first molars are limited to a minimum of four cusps and a maximum of nine cusps. Multiple developmental pathways produce the same numbers of cusps, despite highly variable cusp morphologies, indicating the existence of limits on how morphological evolution can fill a morphospace defined by cusp numbers. These constraints are both developmental and phylogenetic in nature and the identification of their influence on rodent molar shape provides a framework for investigation of how tooth batteries evolved an array of functions despite fundamental structural limits. The data presented here increase predictability of cusp number and evolutionary outcomes of rodent cheek dentition.

## Introduction

Mammal teeth show great diversity in morphology and function, which is directly correlated with dietary ecology and evolution^1–3^. Convergence is a major feature of mammalian tooth evolution, but the mechanisms underlying this homoplasy are unclear. For instance, function and dietary ecology alone are insufficient to explain phenotypic convergence because convergent phenotypes appear to share structural components that are thought to be homologous^1^. To understand convergence in any morphology, it is necessary to understand the constraints that affect it. Here, I investigate constraints on a major structural feature of tooth morphology in rodents, cusp number. I use an *in silico* modeling approach and empirical data from a broad sample of rodents to determine these constraints. In the course of identifying these constraints, I uncover rules that underlie morphogenesis (morphogenetic rules), which appear to be at least as ancient as the clade Glires.

Constraint is most readily recognized in three major forms; functional, developmental, and phylogenetic. Functional constraints exist where the phenotype interacts with the environment and are typically revealed through the study of functional morphology. Developmental and phylogenetic constraints are more difficult to separate, but clear definitions, are available, the ones I use here are *sensu*^4^. A developmental constraint exists when it is embryologically possible to make a specific phenotype but genesis of the phenotype results in a fatal outcome for the organism. A phylogenetic constraint is where the ability to make a specific phenotype has not yet evolved or has been lost; therefore the organism has no mechanistic way of producing the phenotype.

It is possible to begin to differentiate developmental and phylogenetic constraints from functional ones by constructing a theoretical morphospace based on morphogenetic interactions, and then comparing empirical data to determine where morphologies overlap^4^. Here, I construct a theoretical morphospace for rodent molars using morphogenetic interactions modeled *in silico*. When compared to the reported empirical data, this morphospace allows the identification of potential developmental or morphogenetic constraints on cusp number in rodents.

Rodents are the most taxonomically diverse group of living mammals, encompassing some 40% of extant mammalian species. They are characterized by a wide range of functionally specialized tooth forms, contributing to the success of their ecological radiations^1^. Substantial previous work has focused on detailing the process by which mammal teeth (in particular rodent teeth) develop embryologically, including the construction of ‘staging’ schemes that demarcate the emergence of the substantial morphological features of a tooth (see^5^: for review). Research on rodent tooth development provides an understanding of how tooth structures form and are constrained by interactions between developing morphological structures and the genetics that underlie them (e.g., ^6,7^).

Rodent odontogenesis is broadly characterized by five main stages that follow one another in embryonic days^5^. First, the ‘thickening’ stage (embryonic day 12.5 or E12.5) is when an invagination of the dental epithelium becomes visible. Second, the “bud” stage (E13.5), where the invagination of the dental epithelium has formed a bud-like structure and condensing neural-crest based mesenchyme is present at the base of this bud (the tooth is referred to a bud from this point until it erupts). Third, the “cap” stage (E14.5) is where the mesenchyme has sufficiently condensed that the bud is now capped by one or several primary enamel knots. Fourth, the “early bell” stage (E16.5): here further expansion of the epithelium into the mesenchyme has occurred and differentiation of tooth layers (inner enamel epithelium, dental papilla, and dental follicle) has begun, along with the differentiation of primary enamel knot(s) into secondary enamel knots on the tooth cap. Fifth, the “late bell” stage (E20), where ameloblasts are now differentiated into the distinct tooth layers commonly recognized (e.g., pulp, dentin, and enamel) and the tooth is ready to erupt. Each of these stages are initiated and limited by a host of genetic factors that control organogenesis and odontogenesis (see^1^: and^5^ for review). It is within these five stages where minute changes to the developmental regime occur most readily and simultaneously produce substantial morphological differences. For example, reorganization of tissues surrounding developing tooth buds that causes less compression of the bud during cap and bell stages results in the characteristic “zig-zag” or “Christmas tree” patterns of vole molars^8^. Because all rodents, and all mammals, share this developmental staging, the basic structures of mammalian teeth are homologous. This fact is of critical importance when we investigate the changes to these structures that result in distinct tooth morphologies (and functions).

Empirical observations of cusp numbers in the fossil record show apparent trends characterized by the addition of cusps on the anterior end of the lower first molar in distantly related rodents such as voles^9^; muskrats^10^; and caviomorphs^11^. These cusp additions are homoplastic, but consistent in their location, suggesting that they are constrained in some way. Constraint can take multiple forms, developmental, morphogenetic, or functional, and disentangling one type of constraint from another is exceptionally difficult. This is because constraints are fundamentally linked to one another. For example, developmental and morphogenetic constraints can be thought of as the product of the interactions between *morphogenetic rules* (*sensu*^12^). In turn, morphogenetic rules are simply the *interactions* between two or more morphology-forming genetic controls, which function to produce a *limited set of morphologies* from what is otherwise an infinite morphospace (in other words, morphogenetic rules define theoretical morphospaces^4,12^). Identification of developmental or morphogenetic constraints is possible through the construction of a morphogenetic landscape, which is itself a type of theoretical morphospace. This is done by plotting potential (or known) morphogenetic interactions on the X and Y axes and the resulting morphologies on the Z axis; further addition of modeled morphogenetic interactions results in the space becoming hyper-dimensional. The result is a landscape where morphology is defined by the interactions of two or more genetic controls. It is then necessary to compare empirical data to the theoretical morphospace. The resulting phenotypic limitations inferred from this analysis are interpreted to be constraints (morphospace limiting factors).

To understand the role of development in constraining phenotype, and therefore to disentangle developmental and morphogenetic constraint from functional constraint, I used *in silico* modeling to build a morphogenetic landscape based on the interactions of two genetic factors in odontogenesis as it relates to the lower first molars (m1) of rodents. After identifying two potential morphogenetic rules, I used empirical studies of adult rodent cheekteeth to confirm that there is a constraint placed on rodent cheekteeth by either morphogenetic interactions or developmental regimes, independent of function. My approach uses the modeling program ToothMaker^13^, which models aspects of the embryological development of rodent molars, by generating enamel-knot signaling centers on an epithelial-mesenchyme interface (ToothMaker does not model deposition of mineralized tissues). One feature of embryological tooth development that allows prediction of adult tooth morphology is the number of primary and secondary enamel knots (EKs). This is because EKs are the embryological precursors of adult cusps^5,14^ the number of EKs can serve as a proxy for the number of cusps and vice versa. The relationship between numbers of EKs and cusps provides a link between *in silico* modeling and empirical data.

Here, through the use of *in silico* modeling of enamel knots (EKs) and comparison with counts of cusps on empirical samples, I identify two broad morphogenetic rules that help constrain the number of cusps on rodent molars. One rule is identified as a developmental constraint (the minimum number of cusps found on molars), the other is identified as an phylogenetic constraint (the maximum number of cusps found on molars).

## Results

Both *in silico* and empirical data indicate that the lower first molars (m1) are limited to a minimum four cusps and predominantly a maximum of nine cusps, with complete toothrows limited to 28 cusps.

**Figure 1:**
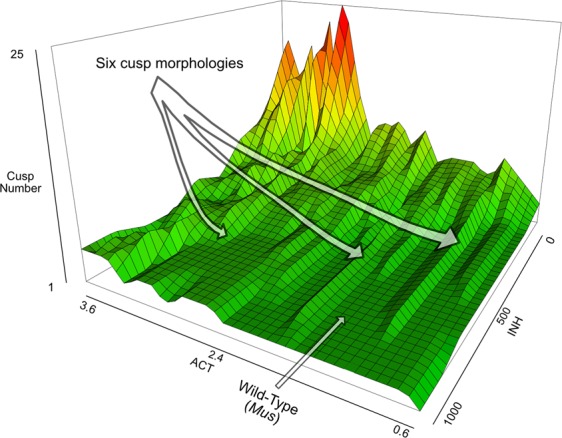
Morphogenetic landscape derived from *in silico* modeling of ACT and INH values. Multiple optima are present that can produce the same phenotype, for instance a six cusp morphology (noted).

*In silico* modeling reveals that increasing initial activator (ACT) concentration over wild-type (WT) conditions can induce additional enamel knots (EKs) on a modeled tooth, but there appears to be a limit of six EKs that can be added by modeling ACT alone (Fig. 1). Doubling of WT-ACT adds one additional cusp (from five to six); Tripling WT-ACT produces what is interpreted as a non-biologically viable tooth (see: Methods, *Biologically ‘non-viable’ models*; Supplemental Information (SI) Fig. 1). Decreasing the initial inhibitory effect (INH) by lowering INH also induces additional cusps, again with a limit of six cusps total before becoming non-viable. Simultaneous manipulation of ACT and INH produces supernumerary cusps, with a maximum number of nine cusps among the biologically-viable models (Fig. 1).

**Figure 2:**
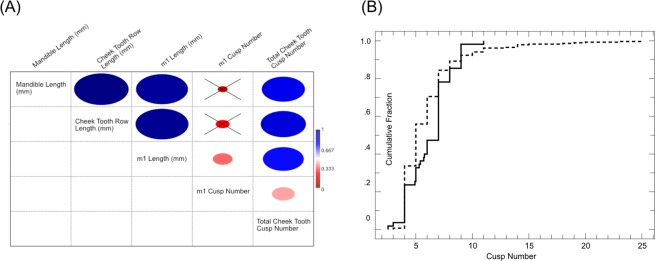
(**A**) Plot of Pearson’s Correlation Coefficient - Size and color of ellipse indicates strength of correlation, crossed out correlations are not significant (P > 0.05), all other correlations are significant. (**B**) Cumulative fraction plot of Kolmogorov-Smirnov test of empirical (solid line) and simulation (dotted line) cusp counts.

*Cusp counts* of cheekteeth of 48 extant and extinct rodent species ranged between 12–28 cusps total in the toothrow (SI Table 1). Lower m1s consistently have 4–9 cusps. To investigate the potential influence of size of the m1 and/or toothrow length on cusp number in extant species, I used a Pearson’s Correlation Coefficient to investigate the relationship between; m1 cusp number and m1 length, m1 cusp number and dentary length, and m1 cusp number and total toothrow length in 37 specimens. The results reveal a weak, significant, positive correlation between m1 cusp number and m1 tooth length (r = 0.35194, P = 0.0254). A weak, non-significant, positive correlation between m1 cusp number and toothrow length (r = 0.24793, P = 0.1392) and m1 cusp number and dentary length (r = 0.17351, P = 0.3198) is also recovered (Fig. 2A). I also investigated the correlation between total cheek-toothrow cusp number and m1 cusp number, to consider if total cheektooth cusp number potentially influenced m1 cusp number; I recovered a weak, significant, positive correlation between m1 cusp number and total cheek-toothrow cusp number (r = 0.41083, P = 0.009). Because length data were not available for fossils, only cusp numbers are reported here; phylogenetically early rodents represented by fossils have similar counts to those in extant species, with estimated total cusp numbers between 12–25, and cusp numbers of m1s between 4–9 (SI Table 1).

## Discussion

The results identify genetic interactions that define some of the morphogenetic rules for rodent (and potentially all mammal) teeth. Genetic interactions that limit enamel knot number embryologically result in a constraint on cusp number in adults. In particular, modeling indicates that there is a minimum of four enamel knots (morphogenetic rule 1) and a maximum limit of nine enamel knots (morphogenetic rule 2) that can be formed on the m1; 84% (n = 205) of simulations recover 4–9 cusps; suggesting that 4–9 cusps on m1 should be the most common form. This result is supported by cusp counts on extant and extinct rodents, where 97% of observed specimens (n = 51) have between 4–9 cusps. The rarity of recovering more than nine cusps in simulations (13%, n = 39) suggests that empirical examples with greater than nine cusps should be rare. Only one taxon in this study sample (*Hydrochoerus hydrochaeris*; capybara) has more than nine cusps present. Biologically viable simulation models with less than four cusps are exceptionally rare (<1%, n = 2), providing an expectation that few, if any, empirical examples will be recovered. Only transgenic *Mus musculus* specimens showed fewer than 4 cusps (n = 2), and both specimens have such low doses of ACT that they are considered effectively non-viable^13^. Comparison of *in silico* and empirical cusp counts using a Kolmogorov-Smirnov test suggests different underlying distributions of cusp counts for the simulation and empirical data (D = 0.2321, P = 0.011). A cumulative fraction plot (Fig. 2B) reveals that the fraction of cusps found in the empirical data is actually less than recovered in simulations, with the maximum deviation between the two occurring between seven and eight cusps. Simulations suggest we should find more empirical specimens with seven and eight cusps than we do; implying that the patterns revealed by the simulations are conservative relative to biological reality.

The identification of minimum and maximum cusp numbers does not explain the variation in cusp number present across rodents. One potential explanation is that the number of enamel knots available for inclusion in m1 is itself variable, and is perhaps driven by the absence and presence of premolars. Previous studies have, controversially, demonstrated that expansion of gene signaling centers localized to the m1 shift anteriorly and include portions of primordial lower fourth premolars (p4) in *Mus*^15^. More recent studies have directly investigated the potential of rescuing these primordial buds and demonstrating that they are locationally homologous to p4s^16^. This suggests that m1s may become more complex by “borrowing” enamel knots from p4 rudimentary buds that are resorbed and never fully develop. Examining the empirical cusp counts between species with p4s present and absent reveals distinct averages for m1 cusp number. Mean m1 cusp number for murids (which lack p4) is 7.48 whereas mean m1 cusp number for non-murids with p4 present is 5.47, a significant difference (Welch’s T-test, P = 0.00008). If m1s are “borrowing” enamel knots from p4s that do not develop, our expectation is that the mean for p4 + m1 would be higher than the m1 mean. This is the case, with the p4 + m1 mean being 10.60 whereas the m1 alone is 7.48, a significant difference (Welch’s T-test, P = 0.00004). Additionally, animals with and without p4s present should have total cusp count means that are different. This is also true: 21.9 with p4, 17.14 without, also a significant difference (Welch’s T-test, P = 0.000038). Additionally, the weak correlation between m1 cusp number and total cheek-toothrow cusp number supports the idea that the p4 + m1 complex may well be functioning as a separate developmental module from the remaining cheekteeth. Together, these observations suggest that variation in enamel knot number for the m1 is not only a function of the morphogenetic factors modeled here, but is also related to the presence/absence of the p4, suggesting that the evolutionary loss of the p4 may be necessary for the m1 to develop more than six cusps in many scenarios.

The evolutionary consequences of the results described here are significant. Rodent lower first molars, despite numerous forms and functions, is fundamentally limited by the minimum (four) and maximum (nine) number of cusps that can be formed embryologically. Many developmental pathways exist to reach the limit of nine cusps; but far fewer surpass this limit, and even fewer allow the formation of less than four cusps. These minima and maxima are a reflection of morphogenetic rules interacting during development which serve to constrain occupied morphospace. These constraints are likely more ancient than crown Rodentia, as evidenced by an apparent hard minimum of four cusps within Glires. It would appear that uni-cuspid cheek teeth, the inferred ancestral condition within Synapsida^1^, are not able to be developed within Glires. This suggests that the evolution of the morphogenetic rules defining the minimum number of cusps predates the origin of Glires some 65 million-plus years ago^17^. The timing of the origin of the morphogenetic rules providing a maximum of nine cusps is less clear, but it appears to have been present for at least a few million years in some lineages. For instance, arvicoline rodents (e.g., muskrats) have evolutionarily “stalled” at nine cusps for more than two million years^10^. Molar cusp count results from this and other studies (e.g., ^18^) suggest that these rules may apply more broadly to many, if not all, crown eutherians; though this remains to be investigated and is beyond the scope of this study. These constraints may also be applicable to more ancient molar crown patterns that evolved convergently in different clades; such as the tribosphenic teeth that underlie the Mesozoic evolution of modern mammal clades^2^.

In this study, it appears that one developmental and one phylogenetic constraint are each present. The constraint on minimum cusp number appears to be developmental because fewer than four cusps is rare in the morphogenetic landscape and only non-viable transgenic *Mus* lines (per^13^) produce fewer than four cusps on the molars. The constraint on maximum cusp number appears to be phylogenetic. While it is uncommon to recover more than nine cusps in the morphogenetic landscape, at least one species, *Hydrochoerus hydrochaeris*, the capybara, does have more than nine cusps present.

Capybara may turn out to be the exception that enforces or “proves” the rule. Recent work sequencing the genome of capybara has discovered high synonymous mutation load, significant protein evolution within the Insulin-Insulin-like or Insulin-growth-factor signaling pathway (IIS/IGF1), and a potentially novel t-cell anti-cancer enhancer that functions on a tumor suppression pathway^19^. The IIS/IGF1 pathway has been identified as playing a role in odontogenesis, including being able to promote Fibroblast-Growth-Factor-4 (FGF4) producing enamel knots in human dental pulp cells^20^. It is also known that the Ectodysplasin-Activator-Ectodysplasin-Receptor (EDA-EDAR) pathway plays a critical role in activating and maintaining a tumor necrosis pathway in mammals^21^. And though entirely speculative at this stage, it is possible that the novel capybara anti-cancer enhancer may increase EDA expression in some fashion. None-the-less within capybara, it appears that the rules are broken likely be the increased expression of IIS and/or potential for a novel anti-cancer enhancer to play a role in influencing enamel knot number and formation. Importantly, it appears that, in the lone case of a rodent exceeding nine cusps on its m1, this exception required substantial regulatory and developmental evolution. Therefore the conclusion is that addition of cusps must be a phylogenetic constraint. The additional data offered by cusp number variance in the presence and absence of a p4 (itself a phylogenetically distinct character) further suggests that the ability to add cusps to the m1 is a function of phylogeny and not only development and thus is a phylogenetic constraint (*sensu*^4^).

It is important to acknowledge that the factors modeled here are a fraction of the genetic factors controlling odontogenesis. The morphogenetic rules identified here indicate that genetic interactions between ACT and INH serve to constrain a portion of the lower first molar (m1) morphology. This is not the sum total of the factors influencing molar morphology or even cusp number (see discussion of the potential role of p4 above). This means that the morphogenetic rules defining cusp number are multidimensional and potentially hierarchical in nature. The rules described here conservatively apply only to the lower first molar of Rodentia. The rules may apply more broadly phylogenetically (e.g., across Glires) and spatially (e.g., the entirety of the lower/mandibular cheekteeth), however this remains to be tested. Importantly, the rules described here do not apply to any teeth outside of the cheekteeth (i.e., incisors or canines). It may seem counter-intuitive that morphogenetic rules can apply to only a narrow anatomical system, but there is currently evidence to suggest that cheekteeth and non-cheekteeth are maintained in separate developmental and evolutionary modules^22^.

## Conclusions

Modeling reveals that multiple developmental pathways produce the same limited numbers of cusps, despite extensive variation in cusp morphologies. These constraints on tooth structure (and by proxy morphospace) are the results of developmentally linked morphogenetic rules that fundamentally limit the number of enamel knots. The rules are minimally, but unlikely totally, defined by the interactions between two genetic factors, the physical concentration of the activator gene(s) (ACT) and the strength of the initial inhibitory effect (INH) of other products that interact with the activator(s).

In identifying morphogenetic rules controlling molar cusp number within Rodentia, I have provided a substantial step forward in the study and application of morphogenetic rules and theoretical morphospaces to studying evolution. This approach facilitates mapping the interactions between genetic factors and their morphological products, and the use of *empirical* data to confirm our findings, an approach advocated by other researchers (e.g.,^4,12^ and^23–26^). Through the identification of morphogenetic rules we can further develop predictive models of morphological evolution.

Future work should focus on investigating the role of other morphogenetic factors, such as the role of buccal and lingual bias on tooth shape and cusp number; and investigation of the mechanistic (genetic and selective) implementation of enamel knot limitations (i.e., how are morphogenetic rules applied?). Rarely are we able to map genotype-to-phenotype, but in the case presented here, we have a compelling starting point. Beginning with the genetic basis of mammalian odontogenesis, there is a short list of potential gene interactions and products to investigate that influence resulting morphology, and study of these in light of the morphogenetic rules identified here should provide insight into how limitations are implemented. The *in silico* modeling approach used here has generated testable hypotheses with respect to how much activator and inhibition effect should be present for any given potential pathway to produce a given morphology within Rodentia and potentially Glires (i.e., researchers can perform experimental applications of values of ACT and INH modeled here). Continued methodological advances in real-time quantification of gene expression and transcriptomes will allow these hypotheses to be tested in the near future. Empirical data collection from across mammalia should also prove insightful, providing context for understanding the phylogenetic depth of the morphogenetic rules identified here and for allowing the identification of potential ‘rule breakers’. ‘Rule breakers’ will, in many respects allow researchers to identify both the limits of morphogenetic rules and points where substantial evolution within the dental system (i.e., both genomic and phenomic) has occurred.

## Methods

### Cusps and cusp counting

Because cusps can serve as a proxy for the number of enamel knots (EKs) during development, counting cusps allows approximate quantification of the number of EKs present in a given tooth during embryological development. I counted cusp numbers and collected measurement data on the adult post-canine dentition of 40 fossil and extant rodent species. Extant specimen data were collected from the Field Museum of Natural History (FMNH) Mammal Collection (specimen numbers provided in Supplemental Dataset 1, photographs showing cusp counts are provided in Supplemental Information (SI)). Fossil data were collected from images associated with publications (specimen numbers and publication citations provided in Supplemental Dataset 1) Additionally, I utilized reported cusp count data from Harjunmaa *et al*.^13^ to add nine species and 17 specimens to my dataset, to provide a total of 57 specimens across 48 species of extant and fossil rodents. This sample represents multiple species from all living rodent families (based on^27^) and the oldest definitive stem Glires^17^ (Supplemental Dataset 1).

### *In silico* modeling

Lower first molars were modeled in ToothMaker (v 0.47) described by Harjunmaa *et al*.^13^ and available for download at: http://www.biocenter.helsinki.fi/bi/evodevo/toothmaker.html. ToothMaker incorporates 26 variable parameters to model embryological development of the lower first molar^13,28,29^. Importantly, ToothMaker models embryological teeth, not adult dentition. Thus size, shape, and occlusal patterns should not be interpreted as representative of adult morphology. Instead, the models represent early stage teeth (stages E12 through ~E20 are modeled). The results of Harjunmaa *et al*.^13^ demonstrate that the modeled teeth are sufficiently late stage that the final number of EKs in a given model should approximate the number of cusps found on the adult lower first molar.

Default parameters in ToothMaker are those designated to represent a wild-type (WT) *Mus musculus* molar, with five enamel knots present (parameters and results Supplemental Data 2; WT designation by^13^). I altered two of the 26 parameters available in ToothMaker, Initial Activator Concentration (ACT) and Inhibitory Effect (INH), to assess the potential lability of EK (i.e., cusp) addition or removal. I varied ACT and INH based on published experimental results of rodents indicating that ACT directly correlates with cusp number. In particular, developmental experiments utilizing various ACT-null lines have rescued cusp morphology only after the application of an activator (e.g.,^13,30^). Overexpression of an activator can produce supernumerary cusps (e.g.,^13,28,29^). Simultaneously, impedance of inhibitor signaling, when an activator is present, will allow supernumerary cusp formation on occlusal surfaces (e.g.^31^). The expectation is that simultaneously varying ACT and INH will produce the highest numbers of supernumerary cusps in models.

I applied three schemes for altering parameters: ACT was varied while INH was held constant (at the WT designation), INH was varied while ACT was held constant (at the WT designation), and ACT and INH were varied simultaneously. To determine how sensitive the model was to minor perturbations, I adjusted ACT and INH individually and counted resulting enamel knots.

For ACT, I compared the results of adjusting ACT in 0.05 unit increments versus 0.1 unit increments, and 0.1 versus 0.2. I found no difference between 0.05 and 0.1, but found that increases of 0.2 resulted in an averaging of changes, which did not show some of the movements back and forth between enamel knot numbers. For the final analyses, I increased ACT in 0.1 unit increments from 0.5 to 3.6.

For INH, I adjusted by increments of 50 units and 100 units. No difference was found between these two increments, except at very low levels (<300) of INH. Below this point, INH produces teeth that are interpreted as not biologically realistic (SI Fig. 1).

With my sampling schemes established, each model was run for 14000 iterations and all other parameters were default as defined by Harjunmaa *et al*.^13^. The resulting morphogenetic landscape is shown in Fig. 1, a table of results and ACT-INH values is provided in Supplemental Dataset 2.

### Biologically ‘non-viable’ models

I made efforts to explore the limitations of ToothMaker models and as a result identify several ranges which I interpret as ‘biologically non-viable’. Increasing ACT or decreasing INH individually can induce seven or more supernumary cusps, but these models are interpreted as non- viable. Non-viabilty is assumed because the initial domain of ACT does not differentiate into additional EKs during any of the 14000 iterations at higher ACT levels (>4.0) (Supplemental Fig. 1A) or lower INH levels (<300) (SI Fig. 1B), nor do they differentiate if the simulation is run longer (i.e., 28000 iterations) (SI Fig. 1C). The lack of differentiation would, if biologically viable, produce a tooth with a single, large, centrally-located cusp surrounded by a sea of undifferentiated enamel (e.g., SI Fig. 1C). Simultaneous manipulation of high ACT levels (>4.0) and low INH levels (<300) run for a standard 14000 iterations produces the same result as running for 28000 iterations (i.e., SI Fig. 1C) It is also possible that at high ACT/low INH levels the mathematical model underlying these ToothMaker parameters is not capable of differenting this initial domain.

### Statistical analyses

To investigate the correlation between empirical m1 cusp number and length variables, I performed a series of Pearson’s Correlation Coefficient analyses. Pearson’s Correlation Coefficient uses a linear model to compare the correlation between two variables and outputs an r value between 0 and 1, with 0 being no correlation between variables and 1 being complete correlation between variables. Higher r values between two variables are interpreted as being stronger, with lowers values being “weak”. In addition, P-values are provided to determine if “weak” correlations are non-significant. Here, I use a 95% confidence interval (P < 0.05 is significant). In addition to comparison of empirical m1 cusp number and length variables, I investigated whether or not m1 cusp numbers collected from the empirical dataset and those from *in silico* simulations are drawn from the same distribution using a two-sample Kolmogorov-Smirnov test. The two-sample Kolmogorov-Smirnov test outputs two critical values, a D value, which is the maximum vertical deviation between two samples, and a P value, which informs us if the D value is statistically significant. A significant result, allows us to reject the null hypothesis that both samples are drawn from the same distribution.

## Supplementary information

Dataset 1

Dataset 2

Supplemental Information
