# Supplementary material for: Phylogenetic and Developmental Constraints Dictate the Number of Cusps on Molars in Rodents
Source: Sci Rep. 2019 Jul 29;9:10902. doi: 10.1038/s41598-019-47469-x (PMC6662684; doi:10.1038/s41598-019-47469-x)
Supplement: Supplementary file 3 — Supplemental Information [file 41598_2019_47469_MOESM3_ESM.pdf]

Supplementary Information (SI) Belonging To

Phylogenetic And Developmental Constraints Dictate the Number of Cusps on Molars in Rodents

Robert W. Burroughs

Includes:

Supplementary Figure 1
Supplementary Table 1
Supplementary Figures Demonstrating Empirical Cusp Counts for 30 taxa

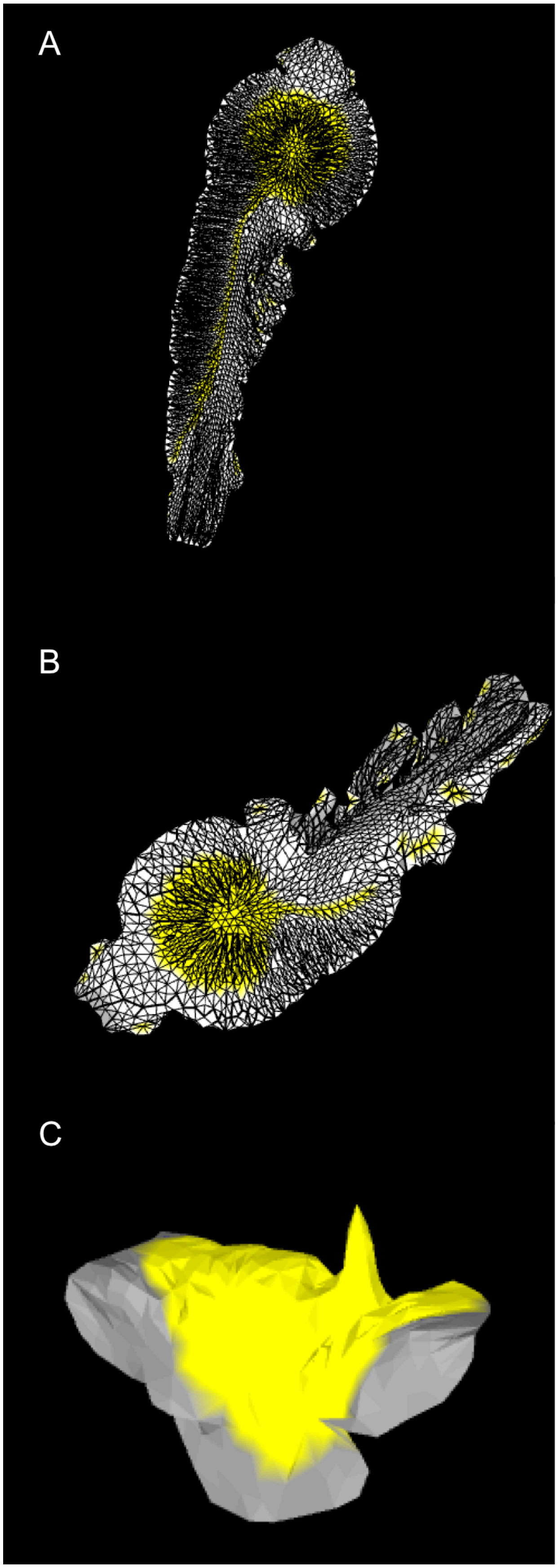

Supplemental Figure 1:

(A) “Tooth” from *in silico* modeling with 4.0 ACT and 800 INH values.

(B) “Tooth” from *in silico* modeling using 1.6 ACT and 160 INH values

(C) “Tooth” from *in silico* modeling with 4.0 ACT, 160 INH, and run for 28000 iterations.

Supplemental Table 1: Sampled species and specimens for empirical cusp counts. Fossils denoted by †. Specimens marked with * are data from Harjunmaa et al. 2014.

**Species/Specimen**	**m1 cusp number**	**Tooth Row Cusp Number (Minimum estimate)**	**Maximum straight line m1 length (mm)**	**Maximum straight line Tooth Row Length (mm)**
Castor canadensis	6	23	6.77	27.82
Cavia porcellus	5	19	3.1	13.4
Hydrochoerus hydrochaeris	11	42	17.74	86
Sciurus niger	5	17	2.71	12.14
Marmota flaviventris sierare	4	17	3.72	18.41
Tamiasciurus hudsonicus richardsoni	4	20	1.6	8.8
Tamiasciurus richardsoni	4	20	2	9.1
Petaurista alboufus candidulus	9	28	4	17.5
Petaurista alborufus lena	7	27	3.5	17.1
Iomys horsfieldi	4	17	1.6	9
Callosciurus atrodorsalis	4	18	1.8	9.2
Tamiops swinhoei spencei	4	19	2.2	6.9
Erethizon myops	7	21	5.7	29.22
Lepus californicus	4	20	3.19	17.5
Sylvilagus flordianus	4	21	2.08	13.78
Protophiomys tunisiensis†	6	23	1.63	6.36
Gomphos sp.†	4	19	NA	NA
Mimolagus aurorae†	NA	23	NA	NA

Aethomys hindei	9	9	2.4	6.2
Apodemus agrarius	8	7	0.8	3.8
Arvicanthis niloticus	9	8	2.6	7
Bandicota indica	7	7	3	9.5
Berylmys bowersi	7	8	3.1	8.7
Chiropodomys gliroides	9	9	1.3	4
Crateromys schadenbergi	7	7	6.3	16.3
Dasymys incomtus	8	6	2.2	6.7
Dephomys defua	7	7	1.7	5.5
Grammomys dolichurus	9	9	1.5	4.4
Hybomys univittatus	8	8	2.4	5.7
Hylomyscus stella	8	8	1.7	3.6
Lemniscomys striatus	9	9	1.8	4.8
Leopoldamys sabanus	7	7	3.7	9.2
Malacomys longipes	7	7	2.4	5.5
Mastomys natalensis	7	7	1.8	4.7
Mus musculus	7	6	1.3	3
Nesokia indica	5	6	2.4	7.6
Notomys mitchellii	7	7	2.1	4.6
Oenomys hypoxanthus	7	9	2.7	6.5
Pelomys campanae	6	6	2.1	5.3
Eda null (0 ng/ml EDA)*	2.54	NA	NA	NA
Eda null (2.5 ng/ml EDA)*	3	NA	NA	NA

Eda null (10 ng/ml EDA)*	4	NA	NA	NA
Eda null (25 ng/ml EDA)*	4	NA	NA	NA
Eda null (50 ng/ml EDA)*	4.92	NA	NA	NA
Eda null (100 ng/ml EDA)*	5.29	NA	NA	NA
Eda null (500 ng/ml EDA)*	5.4	NA	NA	NA
Eda null (1000 ng/ ml EDA)*	5.76	NA	NA	NA
Wild type (0 ng/ml EDA)*	5.7	NA	NA	NA
*Tribosphenomys minutus†**	5	NA	NA	NA
*Anisomys imitator**	7	NA	NA	NA
*Bunomys coelestis**	7	NA	NA	NA
*Hydromys chrysogaster**	6	NA	NA	NA
*Hyomys goliath**	9	NA	NA	NA
*Leptomys elegans**	7	NA	NA	NA
*Mallomys rothschildi**	7	NA	NA	NA
*Niviventer rapit**	7	NA	NA	NA

Supplemental 3: Figures showing cusp counts on extant specimens. All scale bars are 10 mm.

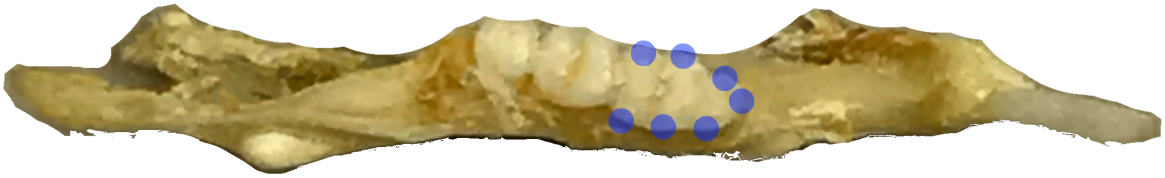

Notomys mitchelli **(FMNH 202351)**

10 mm

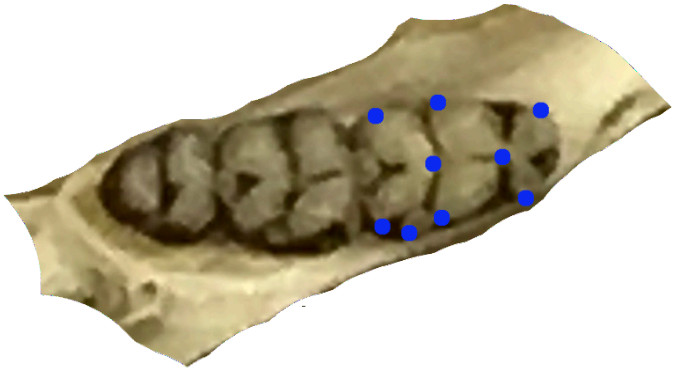

Lemniscomys striatus **(FMNH 231770)**

10 mm

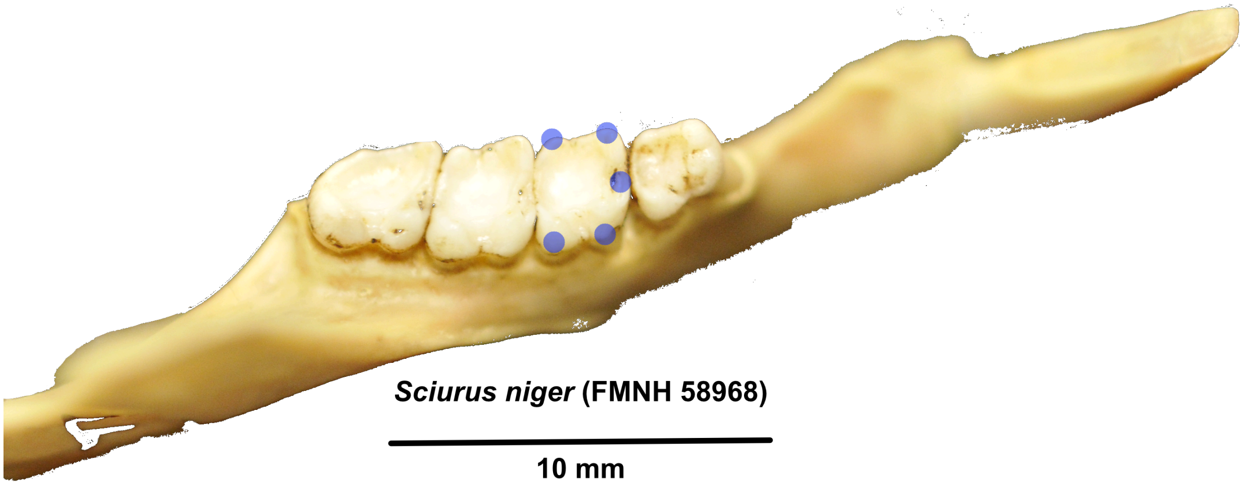

Sciurus niger (FMNH 58968)

10 mm

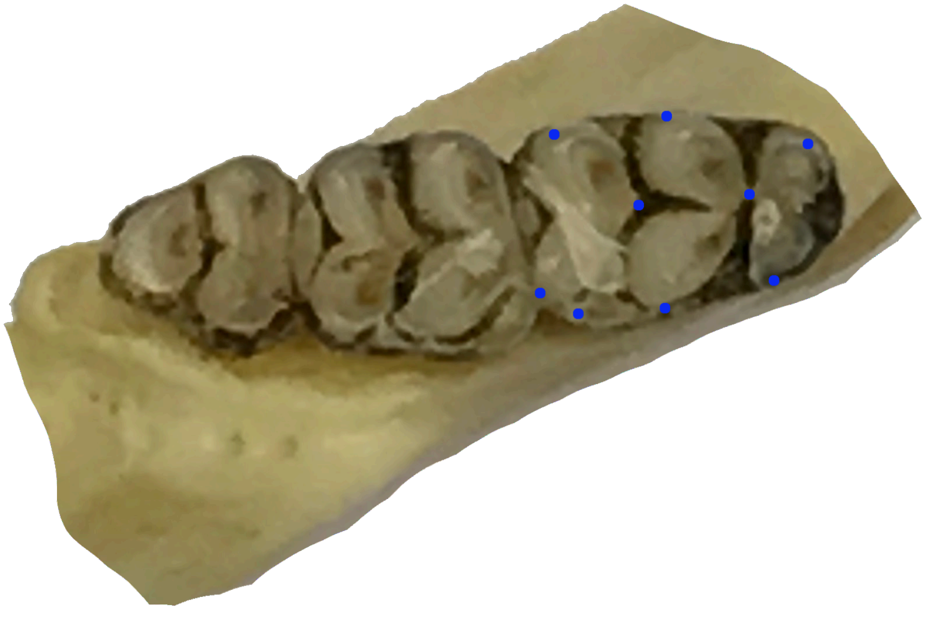

Aethomys hindei (FMNH 153217)

10 mm

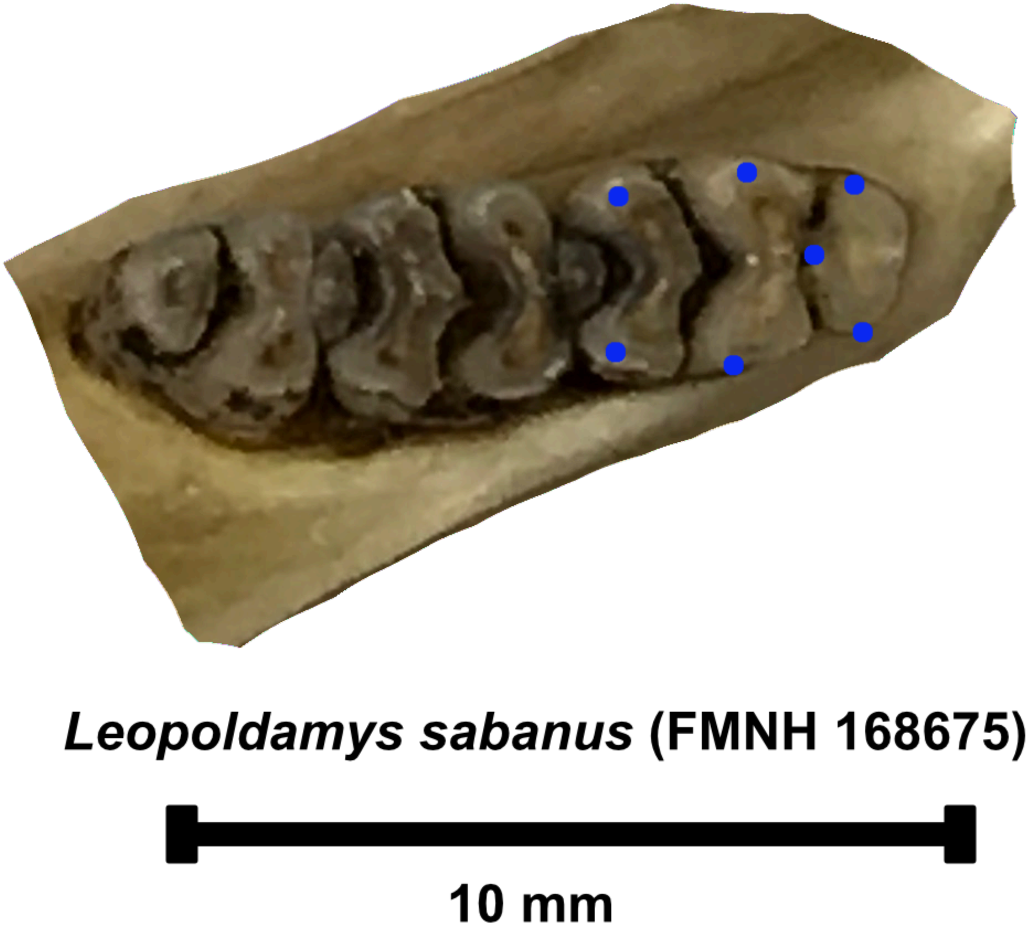
Leopoldamys sabanus (FMNH 168675)
10 mm

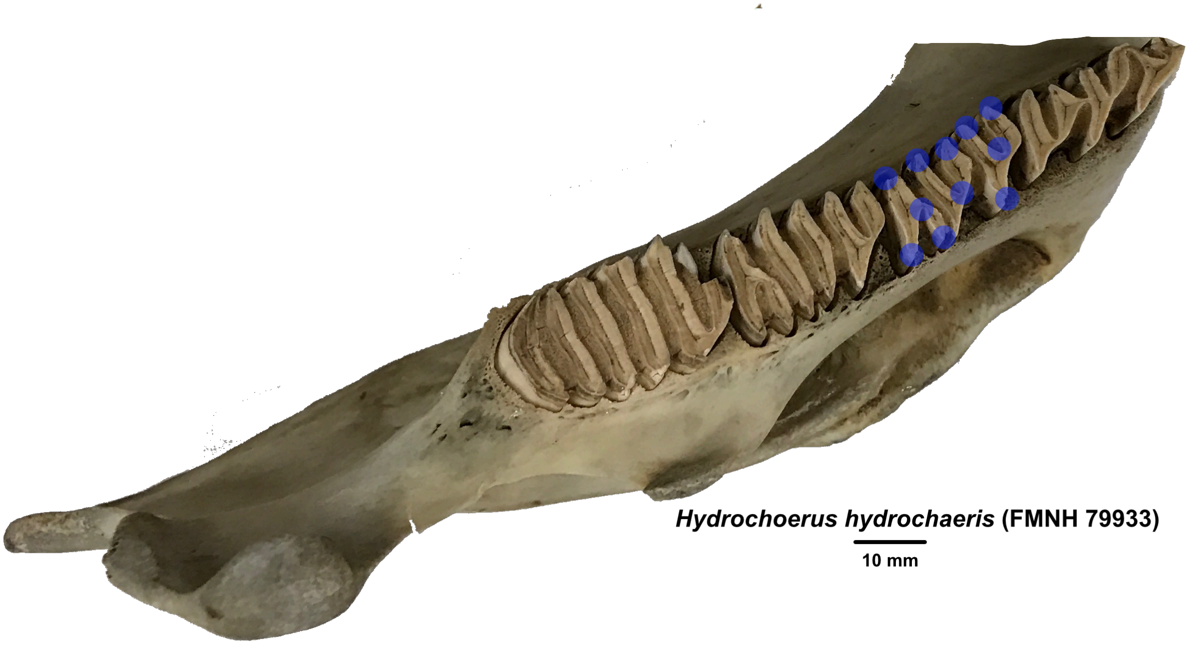
Hydrochoerus hydrochaeris (FMNH 79933)
10 mm

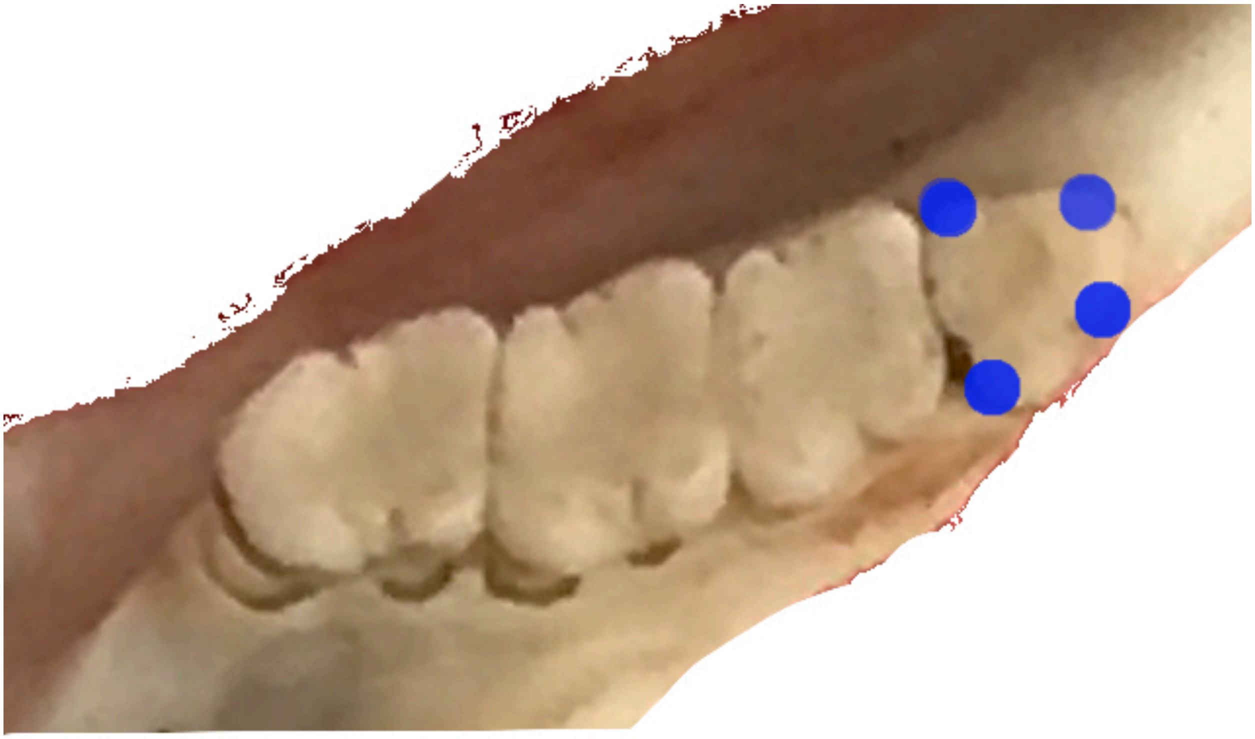

Tamiasciurus hudsonicus richardsoni
(FMNH 90924)

10 mm

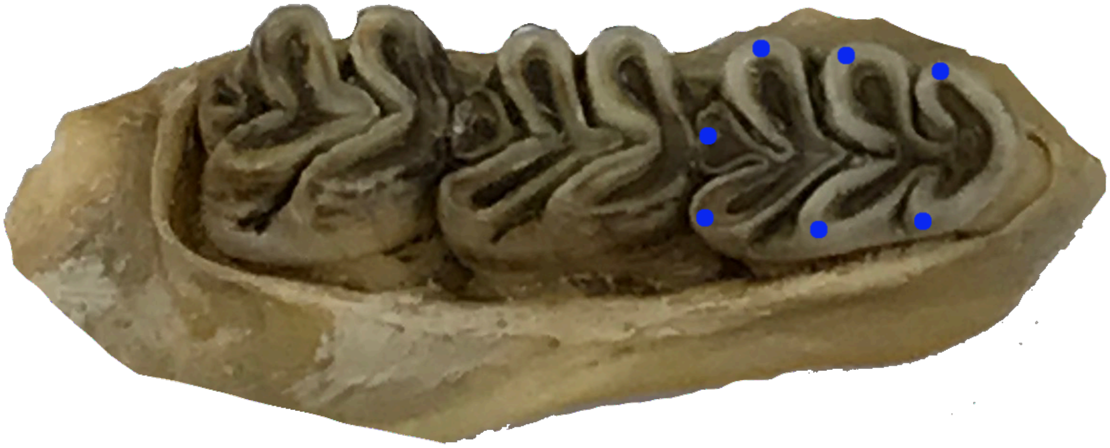

Crateromys schadenbergi (FMNH 62295)

10 mm

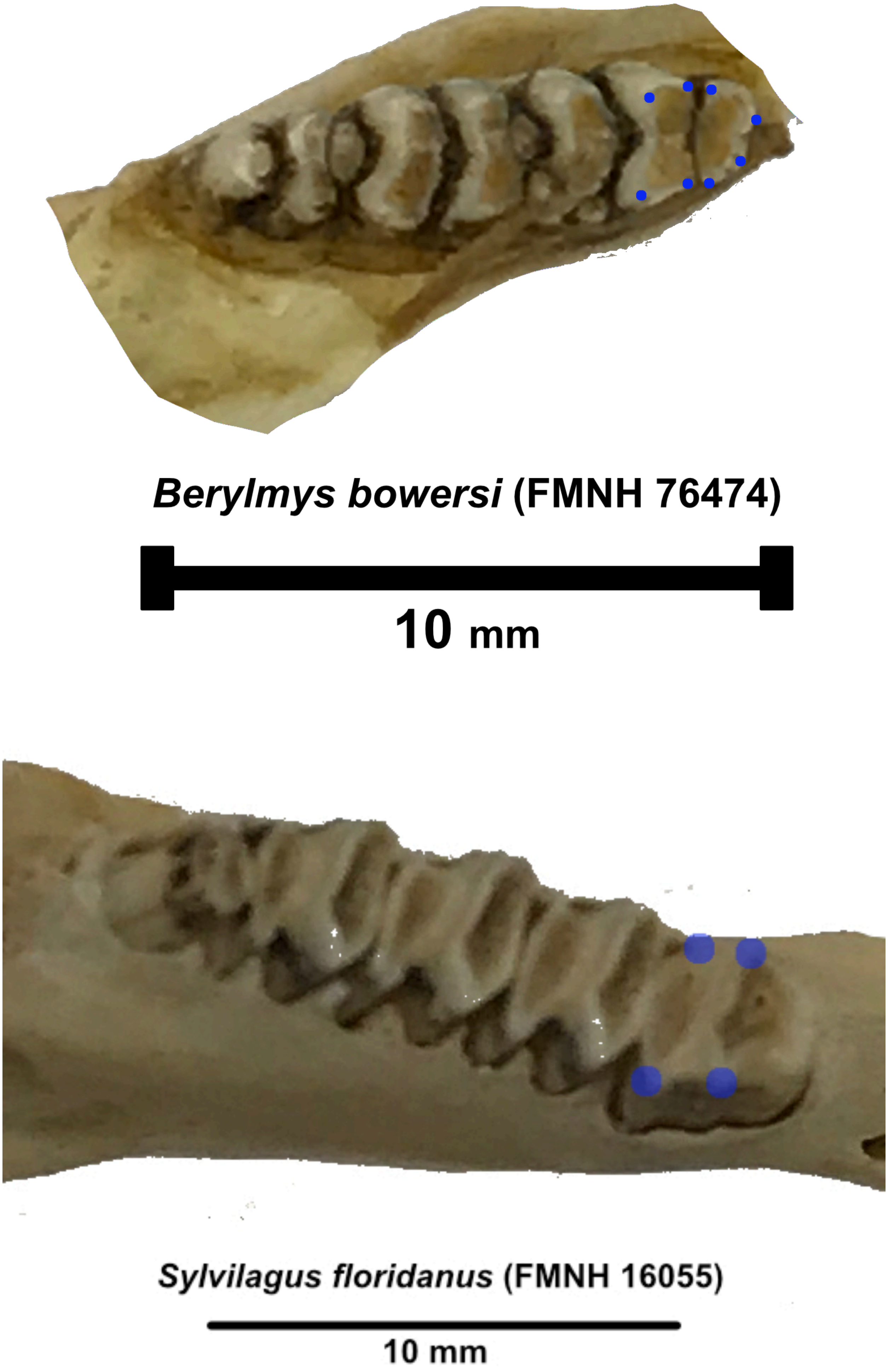
Berylmys bowersi (FMNH 76474)
10 mm
Sylvilagus floridanus (FMNH 16055)
10 mm

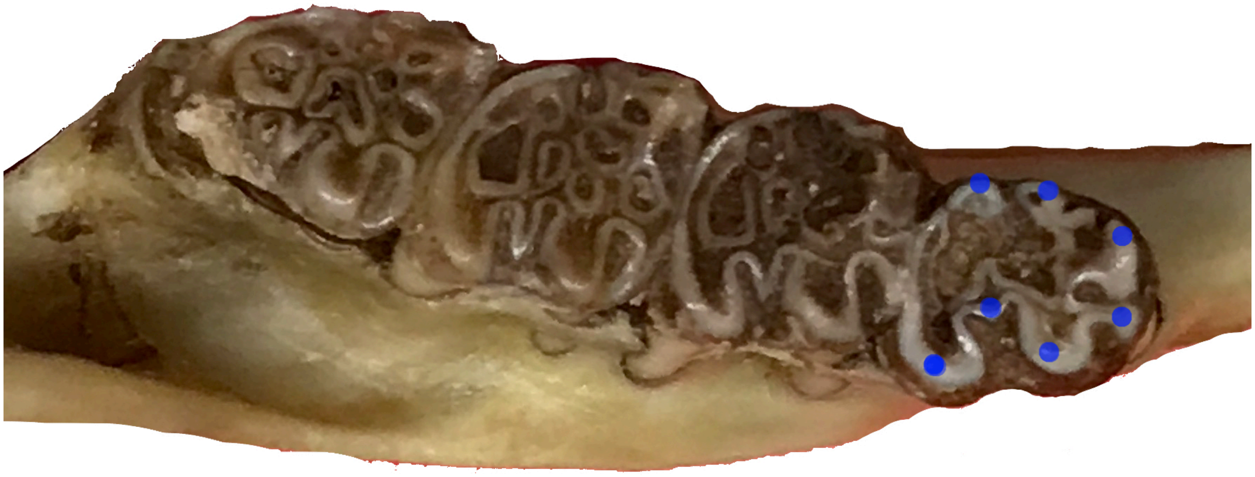

Petaurista alborufus lena **(FMNH 91636)**

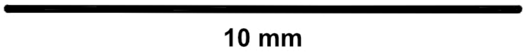

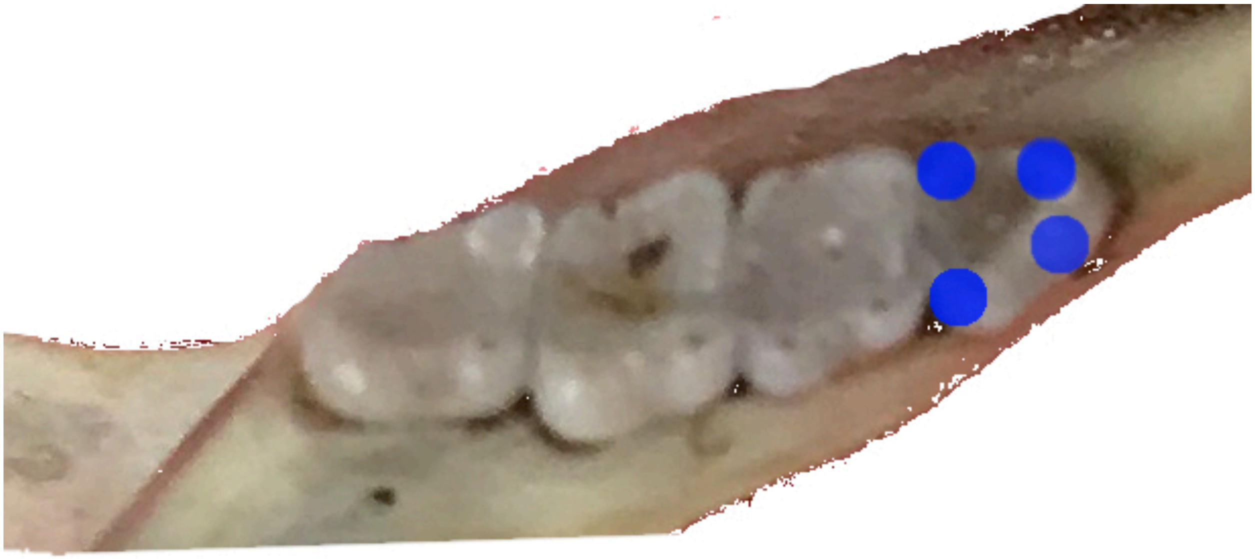

Tamiasciurus richardsoni **(FMNH 5571)**

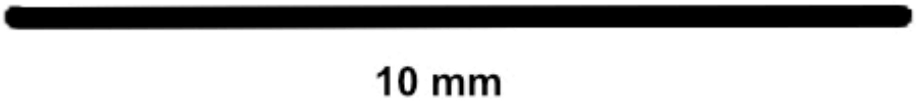

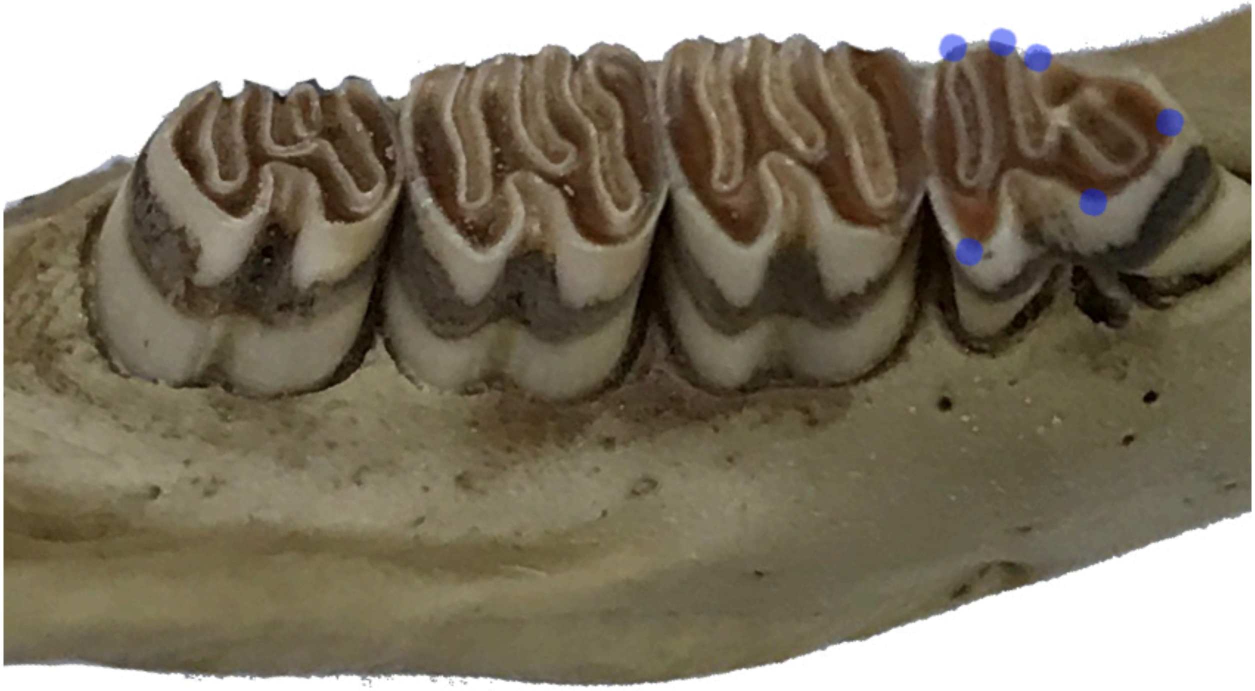

Castor canadensis **(FMNH 134455)**

10 mm

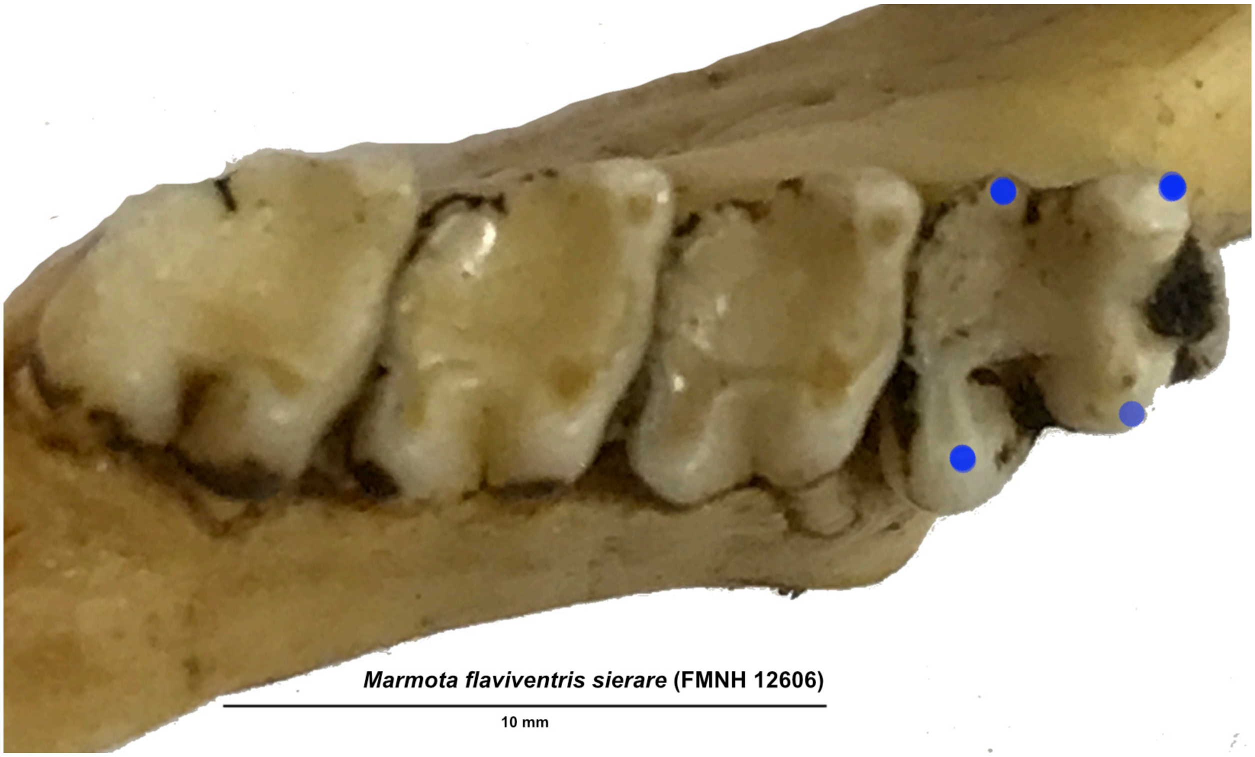

Marmota flaviventris sierare **(FMNH 12606)**

10 mm

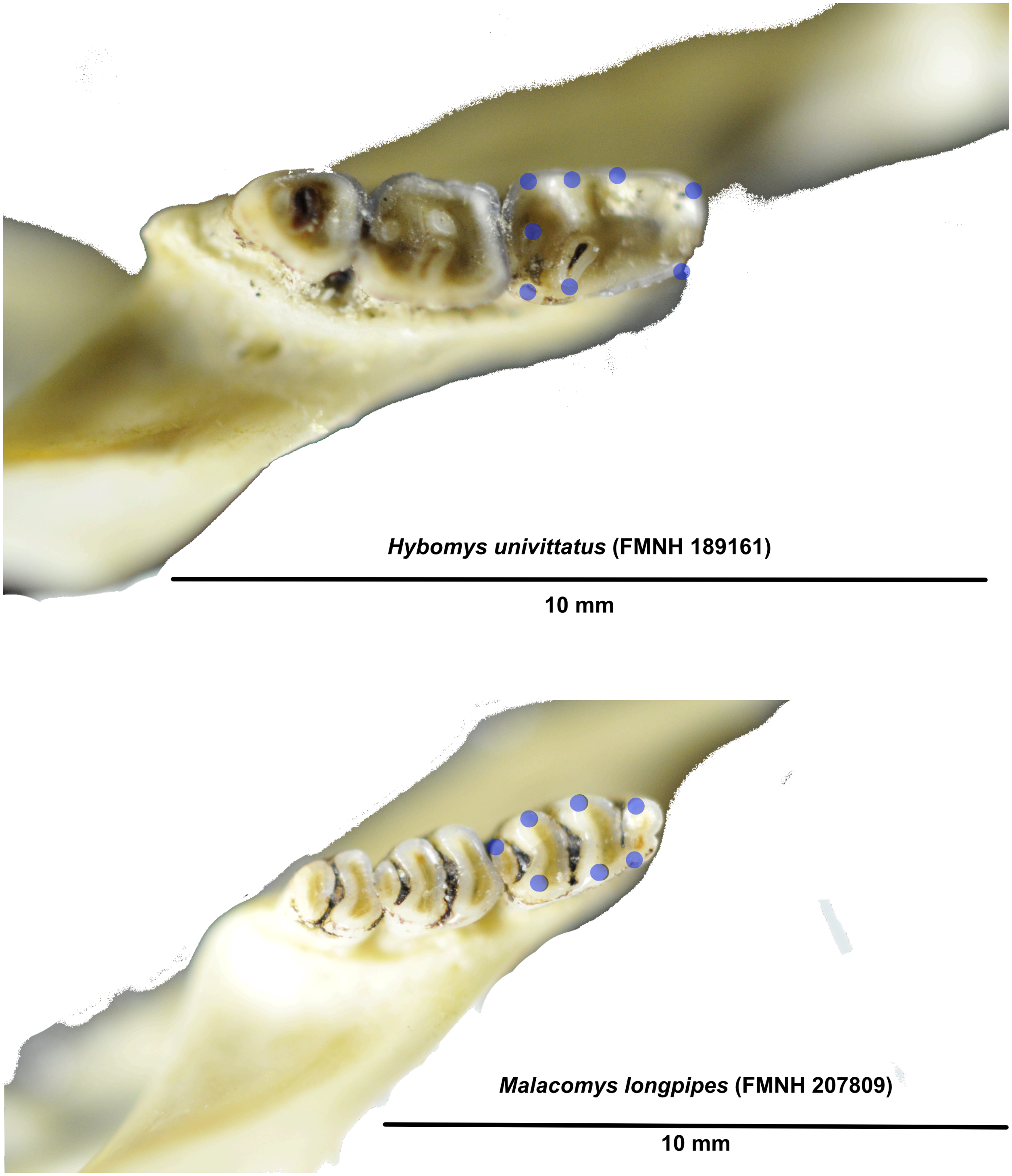
Hybomys univittatus (FMNH 189161)
10 mm
Malacomys longpipes (FMNH 207809)
10 mm

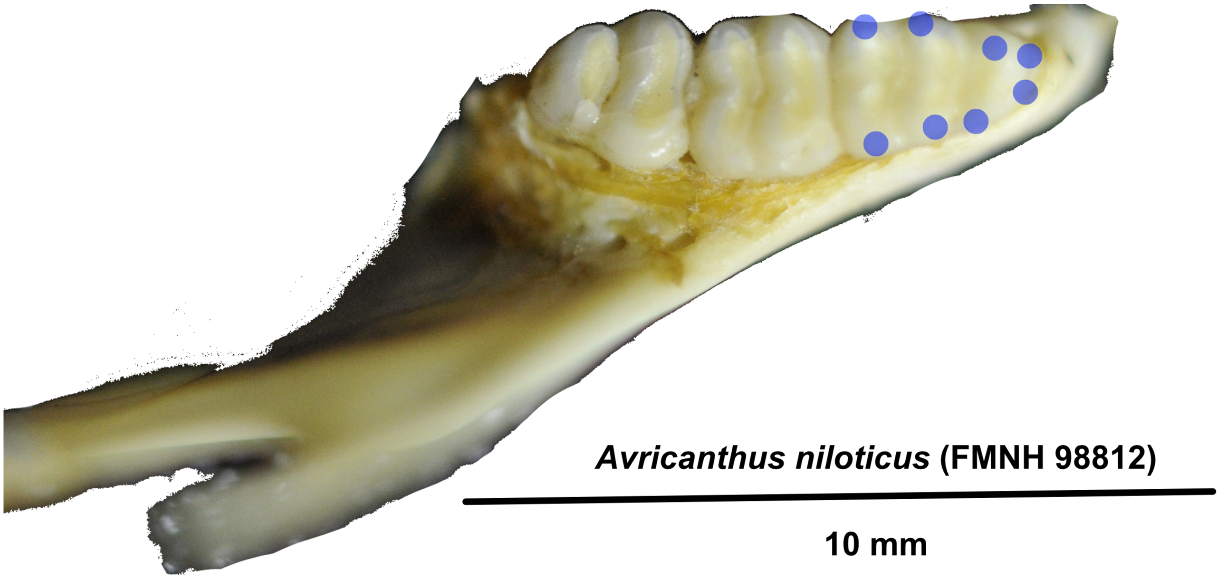
Avricanthus niloticus (FMNH 98812)
10 mm

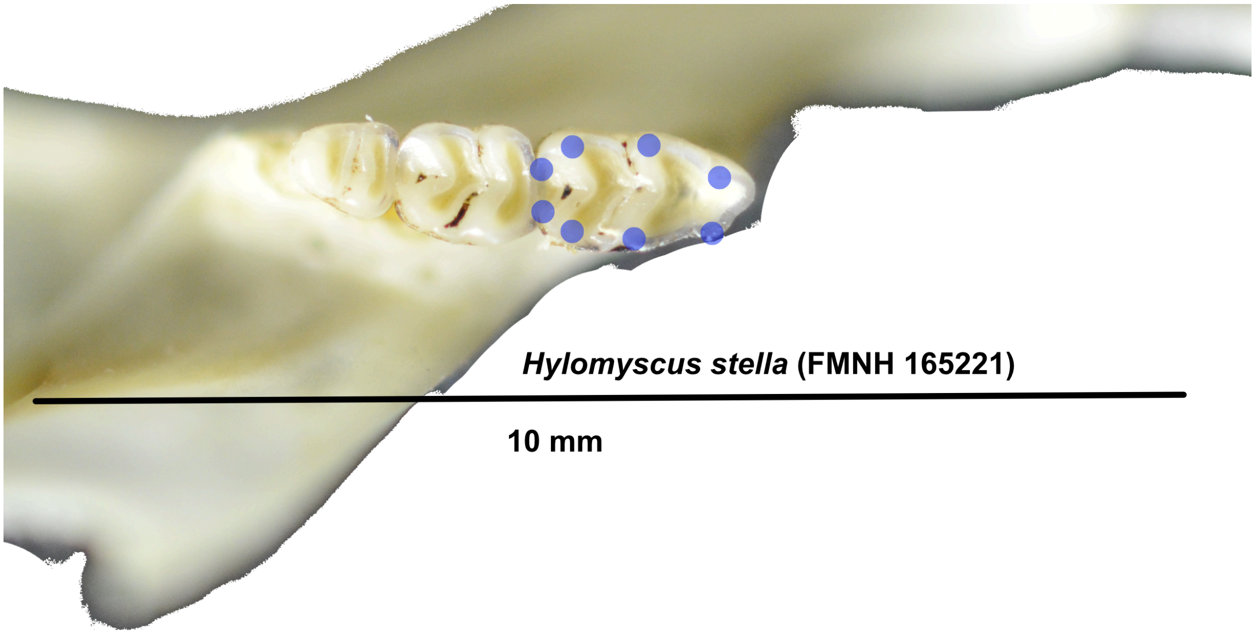
Hylomyscus stella (FMNH 165221)
10 mm

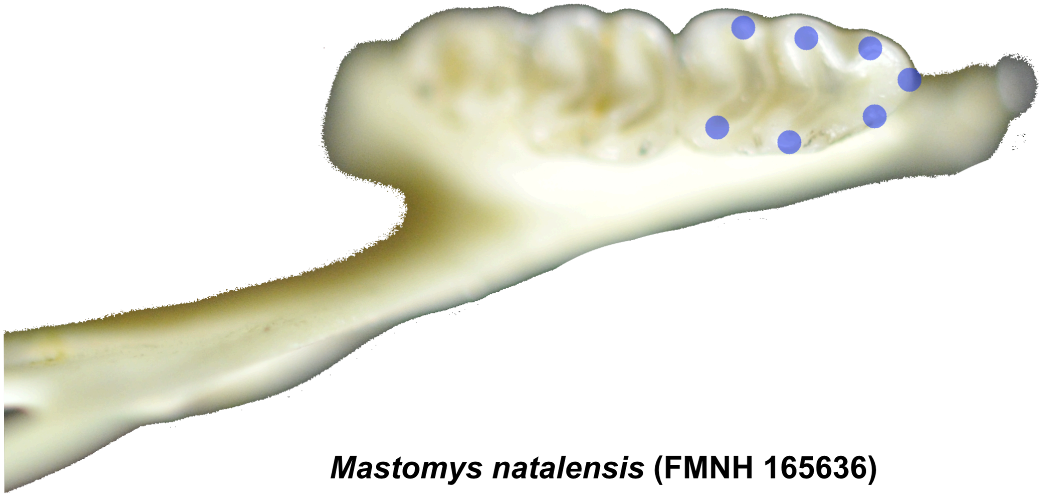

Mastomys natalensis (FMNH 165636)

10 mm

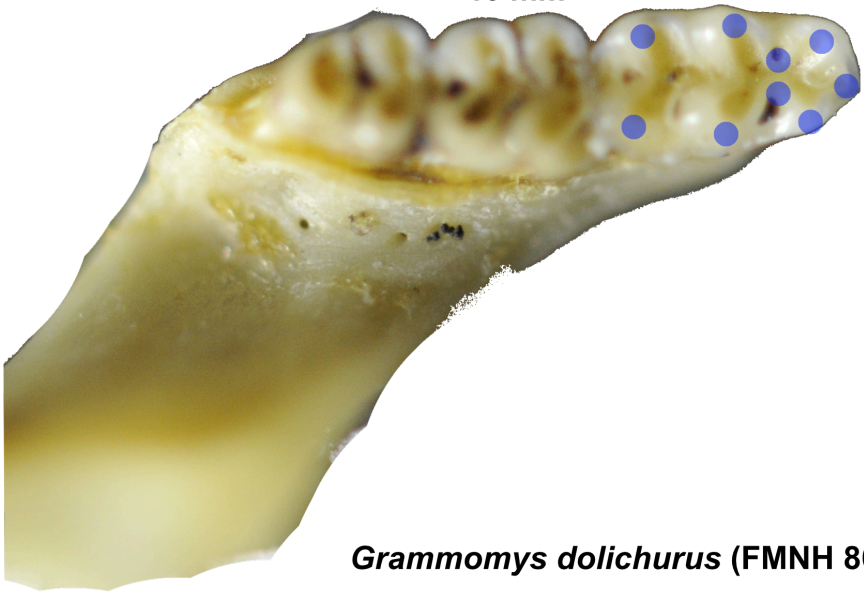

Grammomys dolichurus (FMNH 86252)

10 mm

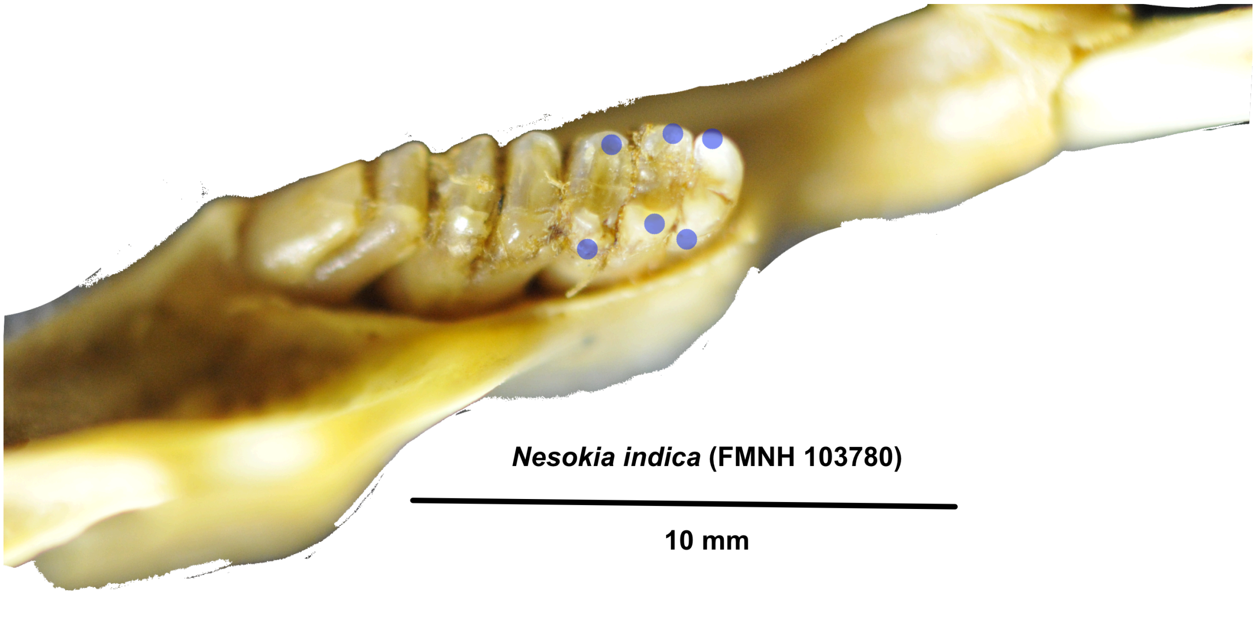

Nesokia indica (FMNH 103780)

10 mm

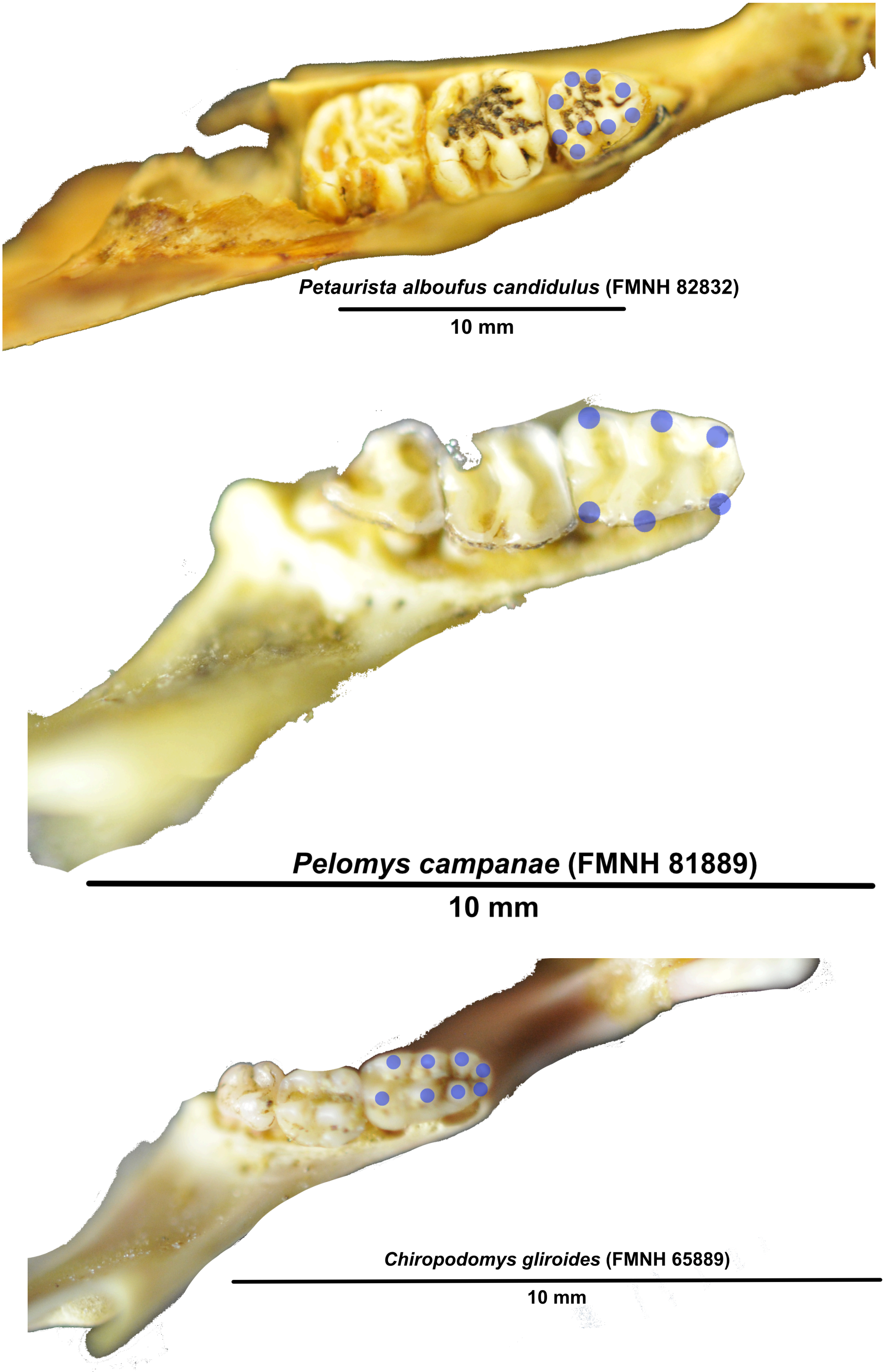
Petaurista alboufus candidulus (FMNH 82832)
10 mm
Pelomys campanae (FMNH 81889)
10 mm
Chiropodomys gliroides (FMNH 65889)
10 mm

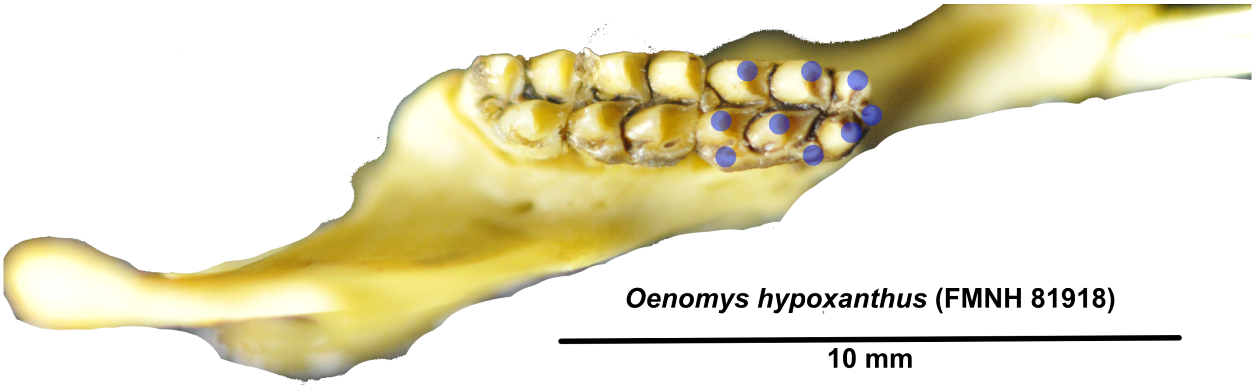

Oenomys hypoxanthus (FMNH 81918)

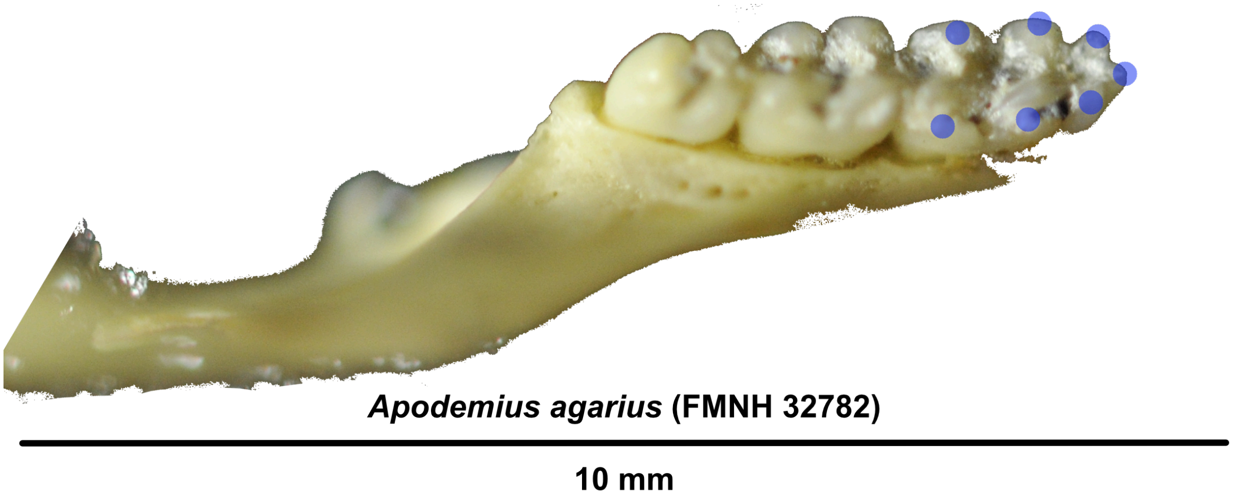

Apodemius agarius (FMNH 32782)

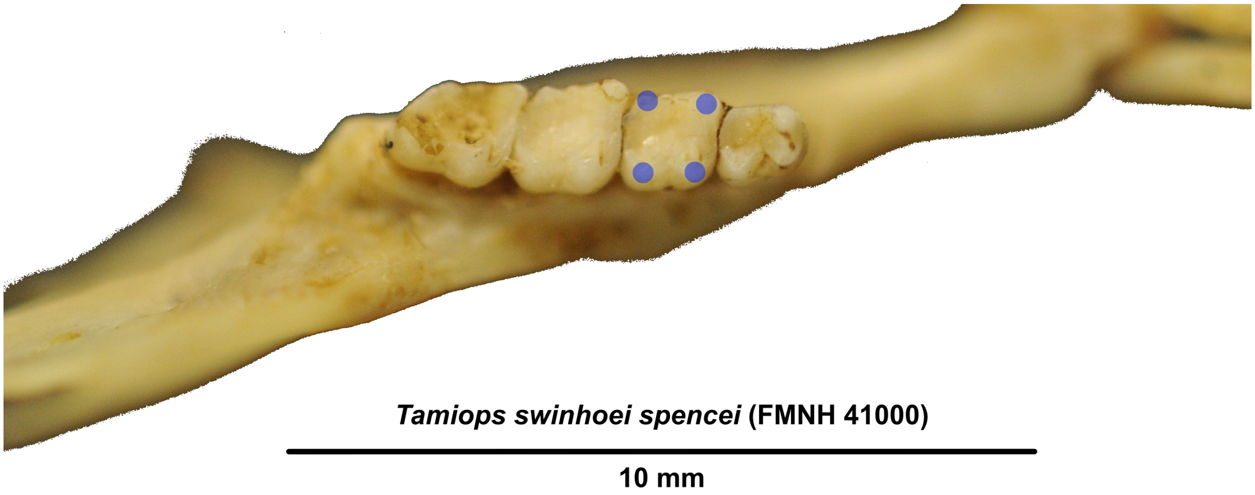

Tamiops swinhoei spencei (FMNH 41000)

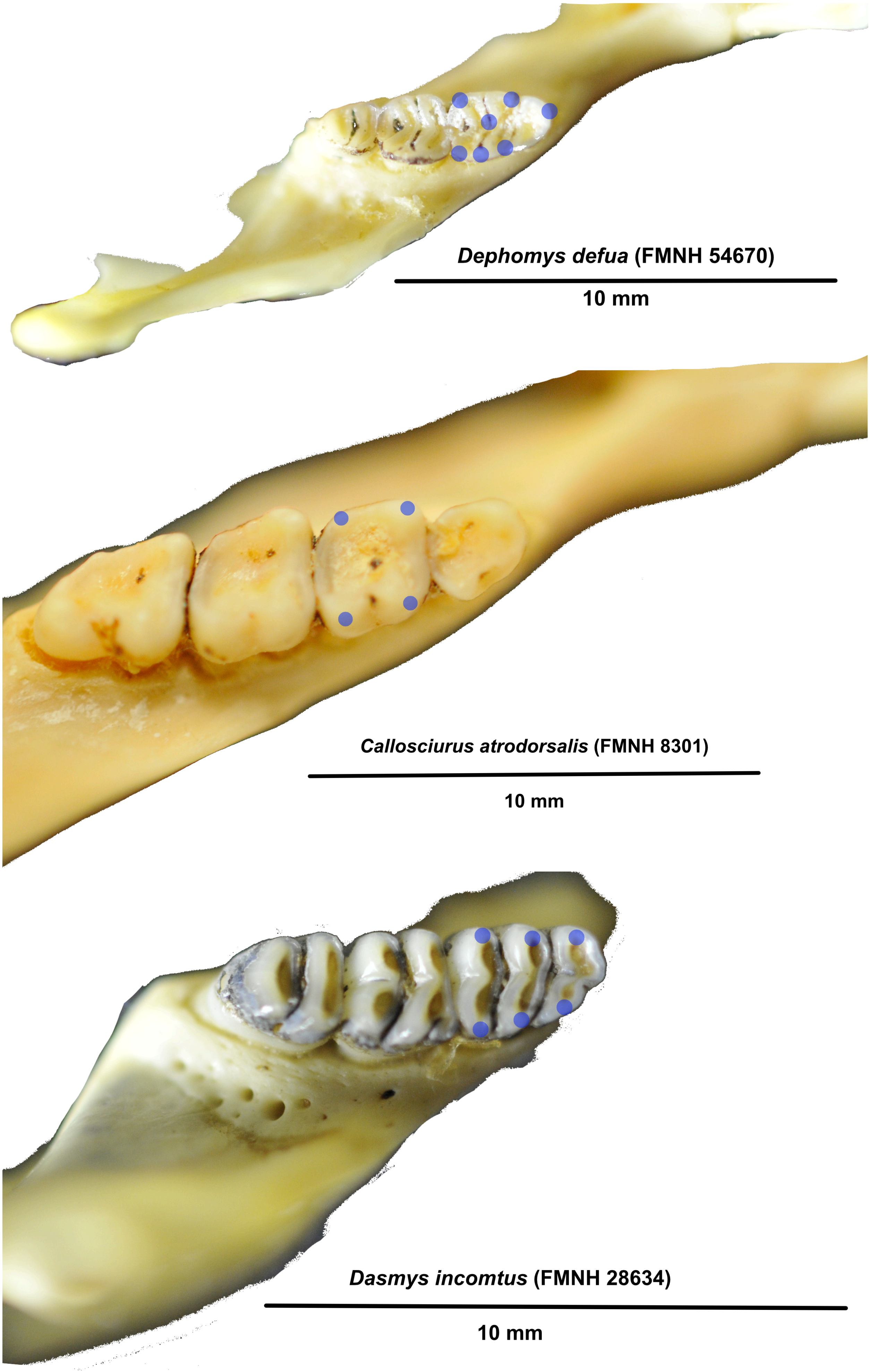
Dephomys defua (FMNH 54670)
10 mm
Callosciurus atrodorsalis (FMNH 8301)
10 mm
Dasmys incomtus (FMNH 28634)
10 mm
